# An Unusual Case of Metastatic Prostate Cancer Presenting as a Massive Urinoma

**DOI:** 10.7759/cureus.41166

**Published:** 2023-06-30

**Authors:** Vikas Dhawan, C P Shanthanu, Sunaina Dhawan, Pathri Manjeera

**Affiliations:** 1 Department of Urology, Indian Naval Hospital Ship (INHS) Asvini, Mumbai, IND; 2 Department of Surgery, Base Hospital, Delhi Cantt (BHDC), New Delhi, IND; 3 Department of Radiology, Shalby Hospital, Mohali, IND; 4 Department of Otorhinolaryngology, Base Hospital, Delhi Cantt (BHDC), New Delhi, IND

**Keywords:** rare clinical presentation, rare presentation, metastatic prostate carcinoma, gleason's score, adenocarcinoma prostate (gleason score 5+5), carcinoma prostate, urinoma

## Abstract

Urinoma formation as the first manifestation of carcinoma prostate is uncommon and has been reported only twice in the literature. We report a case of prostatic carcinoma in an elderly man who first presented in the emergency department with left-sided abdominal pain. His radiological investigations revealed a left-sided urinoma with dilatation of the entire ureter and an enlarged prostate with areas of varied intensity especially in the peripheral zone of the left lobe. In view of raised serum prostate-specific antigen (PSA) (155 ng/ml), a prostate biopsy was done, which showed features of adenocarcinoma (Gleason score 5+5). He was managed with pigtail drainage of urinoma, followed by double J stenting of the left ureter, and later underwent bilateral orchidectomy to reduce tumor burden, being metastatic disease. The stent was removed at a later date.

## Introduction

Urinoma is defined as the extravasation of urine resulting from a discontinuity anywhere in the urinary collecting system and is commonly caused by renal traumas, malignant extrinsic ureteric compression, and urolithiasis in the form of distal ureteric calculi. Urinoma formation as the first manifestation of prostate cancer is uncommon. We report a case of prostatic carcinoma in an elderly man who first presented in the emergency department with urinoma formation. Such an unusual presentation of carcinoma prostate is extremely rare and has just been reported twice in the literature [1,2].

## Case presentation

A 74-year-old male, with no comorbidities, presented at a peripheral hospital with sudden onset severe pain abdomen. On examination, he had tenderness in the left hypochondrium and lumbar region. The patient’s complete blood count and biochemistry were essentially normal. His ultrasound revealed mild hydro-uretero-nephrosis of the left kidney with a perinephric collection extending to the left paracolic gutter. He was managed symptomatically with analgesics and transferred to our hospital.

**Figure 1:**
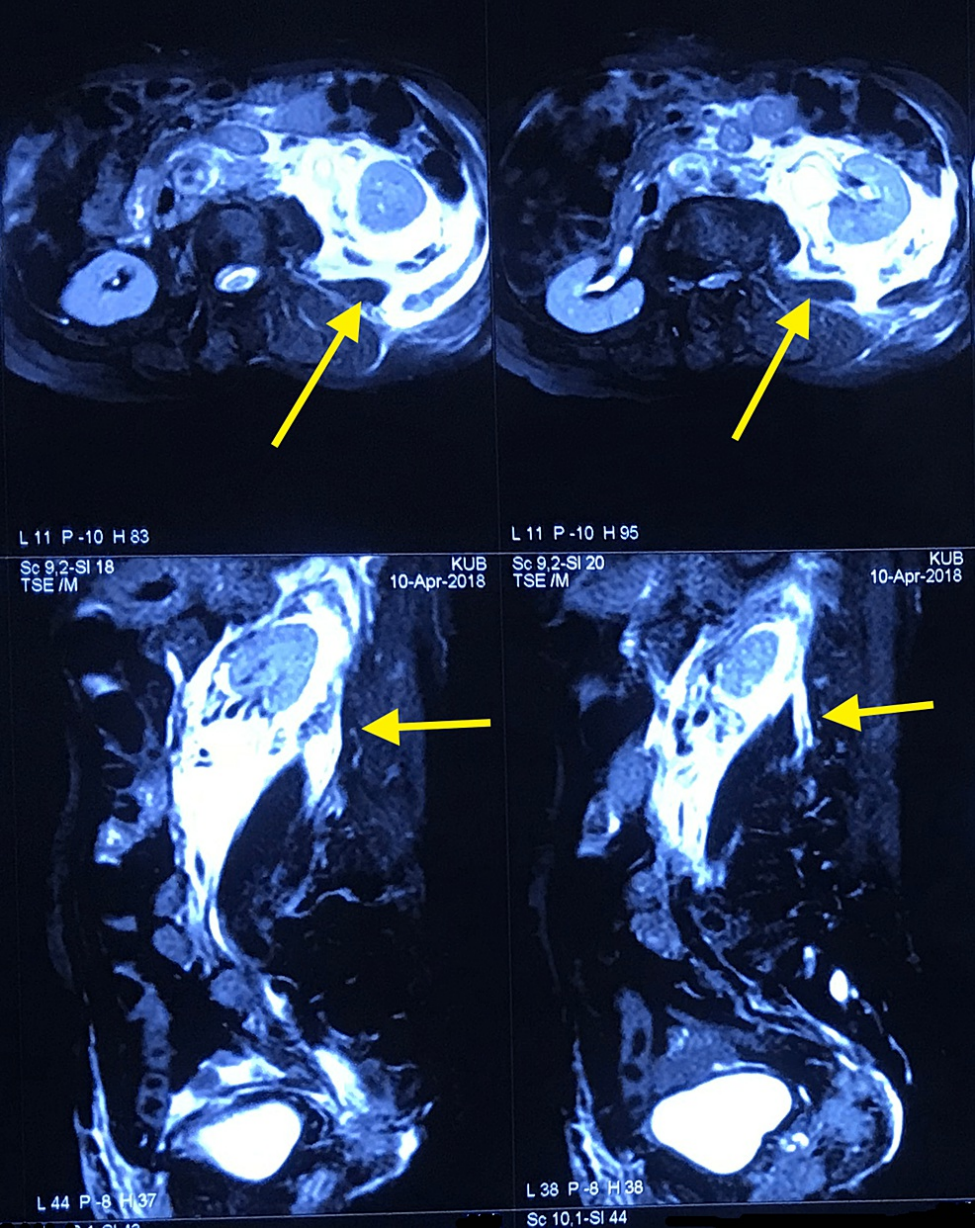
MRI imaging depicting the urinoma

On arrival at our hospital, the patient had persistent left flank tenderness. His digital rectal evaluation (DRE) revealed a grade III hard nodular prostate. His complete blood count and renal parameters were normal. His serum prostate-specific agent (PSA) level was 155 ng/ml. MRI of the patient was suggestive of a well-defined T2-weighted imaging hyperintense collection in the left paranephric space communicating with the inferior pole calyx of the left kidney and extending along the lateral margin of the psoas muscle. The left ureter was mildly dilated in its entire course (Figure 1). The prostate was found to be enlarged with areas of T2 hypointensity with associated restriction of diffusion on DWI images, predominantly noted in the peripheral zone on the left side. The left lobe of the prostate was seen to be extending superioposteriorly to involve the left vesicoureteric junction.

**Figure 2:**
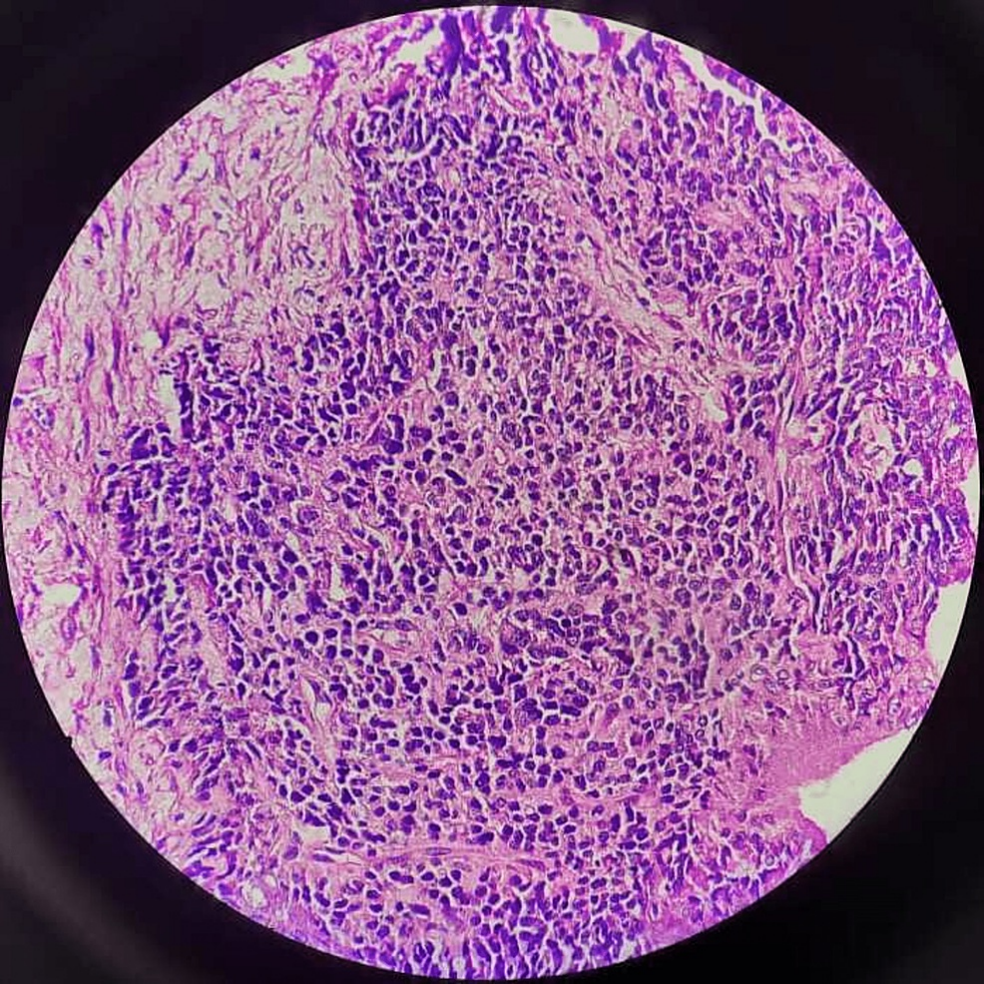
Adenocarcinoma prostate with Gleason score 5+5

The patient was diagnosed with a case of left urinoma. Percutaneous drainage of the urinoma was performed with left pigtail insertion under ultrasound guidance. Approximately 500 ml of clear uriniferous fluid was drained. Subsequently, his drain output remained around 500 ml/day. In view of persistent drain output, he was taken up for left DJ stenting 10 days post pigtail placement. During the procedure, resistance was felt while threading the 5 French size stent over the guidewire at the ureteric orifice. A transrectal ultrasound (TRUS)-guided Tru-cut (core needle) prostatic biopsy was done, which showed adenocarcinoma (Gleason score 5+5) (Figure 2).

Post DJ stent insertion, the patient’s drain output had reduced, and his pigtail catheter was subsequently removed. His bone scan revealed multiple osteoblastic metastases. In view of metastatic carcinoma prostate, he was submitted to bilateral orchiectomy. He was discharged, and the DJ stent was subsequently removed after 90 days, with ultrasound showing resolution of urinoma 

## Discussion

Currently, the majority of prostate cancers are diagnosed based on abnormalities in screening PSA level or findings on DRE. Obstruction due to prostate cancer most commonly presents with upper tract dilatation and impairment without urinoma formation. Urinoma formation as a first manifestation of carcinoma prostate is uncommon. Urinoma is defined as the extravasation of urine, which results from a break or disruption anywhere in the urinary collecting system [3]. Essentially, there are three criteria that must be present or fulfilled for the formation of a urinoma: a functional renal unit, a breach in the pelvicalyceal system, and frequent distal obstruction [4]. The most common causes of a urinoma worldwide are malignant extrinsic ureteric compression, renal trauma, and urolithiasis in the form of distal ureteric calculus [3].

The leaked urine leads to inflammatory changes with lipolysis in the surrounding fat, leading to fibrosis and capsule formation around the collected urine.

CECT/MRI demonstrates the urinoma (size, location) and its relationship to surrounding structures. Delayed scans help demonstrate the exact site of continuity between the urinoma and the urinary collecting system [4]. Image-guided treatment with a combination of percutaneous urinoma drainage catheters, percutaneous nephrostomy catheters, ureteral stents, and bladder drainage is safe and effective [4]. In our case, in spite of percutaneous drainage, there was a persistent high drain output compelling us to put a ureteral stent at a later date.

Urinoma complications include abscess formation, sepsis, urinary peritonitis, paralytic ileus, periureteral and perinephric fibrosis, and electrolyte imbalances [5]. Managing a case of urinoma needs prompt diagnosis and etiology identification, which, if not done, can lead to these life-threatening complications. Urinomas generally resolve spontaneously following treatment or rectification of the underlying obstruction [6].

## Conclusions

We emphasize that radiological imaging plays a very key role in all cases of urinoma presenting in elderly patients in identifying the underlying cause, and carcinoma prostate, though rare, must also be kept as one of the possibilities. Drainage is the universally accepted initial step in the management of a urinoma, and the same principles can be applied to urinomas occurring due to prostate cancers.
